# Line manager training and organizational approaches to supporting well-being

**DOI:** 10.1093/occmed/kqae051

**Published:** 2024-07-11

**Authors:** T Dulal-Arthur, J Hassard, J Bourke, S Roper, M Wishart, V Belt, C Bartle, S Leka, N Pahl, L Thomson, H Blake

**Affiliations:** School of Medicine, University of Nottingham, Nottingham, UK; Queen’s University Business School, Queen’s University Belfast, Belfast, Northern Ireland, UK; Department of Economics, Spatial & Regional Economics Research Centre, Cork University Business School, University College Cork, Cork, Ireland; Enterprise Research Centre, Warwick University Business School, Warwick University, Coventry, UK; Enterprise Research Centre, Warwick University Business School, Warwick University, Coventry, UK; Enterprise Research Centre, Warwick University Business School, Warwick University, Coventry, UK; School of Medicine, University of Nottingham, Nottingham, UK; Centre for Organisational Health and Well-Being, Lancaster University, Lancaster, UK; Society for Occupational Medicine, London, UK; School of Medicine, University of Nottingham, Nottingham, UK; Institute of Mental Health, University of Nottingham, Nottingham, UK; School of Health Sciences, University of Nottingham, Nottingham, UK; NIHR Nottingham Biomedical Research Centre, Nottingham, UK

## Abstract

**Background:**

Employee mental health and well-being (MH&WB) is critical to the productivity and success of organizations. Training line managers (LMs) in mental health plays an important role in protecting and enhancing employee well-being, but its relationship with other MH&WB practices is under-researched.

**Aims:**

To determine whether organizations offering LM training in mental health differ in the adoption of workplace- (i.e. primary/prevention-focused) and worker-directed (including both secondary/resiliency-focused and tertiary/remedial-focused) interventions to those organizations not offering LM training and to explore changes in the proportions of activities offered over time.

**Methods:**

Secondary analysis of enterprise data from computer-assisted telephone interview surveys. The analysis included data from organizations in England across 4 years (2020: *n* = 1900; 2021: *n* = 1551; 2022: *n* = 1904; 2023: *n* = 1902).

**Results:**

Offering LM training in mental health was associated with organizations’ uptake of primary-, secondary-, and tertiary-level MH&WB activities across all 4 years. The proportion of organizations offering primary-, secondary- and tertiary-level interventions increased over time. On average, tertiary-level activities were most adopted (2020: 80%; 2021: 81%; 2022: 84%; 2023: 84%), followed by primary-level activities (2020: 66%; 2021: 72%; 2022: 72%; 2023: 73%) and secondary-level activities (2020: 62%; 2021: 60%; 2022: 61%; 2023: 67%).

**Conclusions:**

Offering LM training in mental health is associated with the adoption of other MH&WB practices by organizations. Suggesting that organizations that are committed to the mental health agenda are more likely to take a holistic approach (including both worker and workplace strategies) to promoting workforce mental health, rather than providing LM training in isolation.

## Teaser

Training line managers (LMs) in workforce mental health has benefits for individuals and organizations, but its relationship with other mental health and well-being (MH&WB) practices is under-researched. Secondary analysis of data collected from enterprise representatives between 2020 and 2023 revealed that organizations offering LM training were more likely to adopt both worker- and workplace-directed MH&WB practices. The adoption of all MH&WB practices increased year on year, with remedial-focused interventions being the most adopted throughout this 4-year period.

## Introduction

National surveys show population declines in personal well-being across the UK [1]. Over the past few years, there has been an increase in mental health challenges among working adults during and after the coronavirus disease 2019 (COVID-19) pandemic [2]. From a public health perspective, the prevention and management of mental ill health through the workplace setting is an important strategy for improving population health [3]. From an economic perspective, for those who are vocationally active, mental ill health is now a leading cause of workplace sickness absence, accounting for around 17 million working days lost each year [4], costing around £56 billion annually [5]. This has implications for the productivity of employees and a high economic impact on organizations [6,7]. Therefore, there are clear public health and economic arguments for promoting mental health and well-being (MH&WB) at work. Despite the rising prevalence of mental ill health, many employers are still unaware of their critical role in supporting the mental health of their employees [5,8], with many employers having limited provisions or policies in place to promote employee psychological well-being [9]. We hypothesize that organizations that offer training to their line managers (LMs) in MH&WB may offer more, or a different profile of, MH&WB policies and practices compared to organizations that do not offer training. Potentially due to an increased awareness and knowledge about workforce well-being amongst their managers who may subsequently implement them. However, there is currently no evidence to demonstrate this.

Workplace mental health interventions are typically categorized as *primary* (prevention-focused activities focused on reducing or better-managing work stressors through job design and management practices), *secondary* (employee-focused activities focused on bolstering their resilience and coping strategies), or *tertiary* (remedial- and curative-focused activities) [10]. A holistic approach integrating all three levels of intervention, which targets both workplace- (e.g. primary) and worker-directed strategies (e.g. secondary and tertiary interventions), is advocated as the best practice [11,12]. However, primary prevention is of particular importance to maximize employee health and productivity [12,13]. The importance of prevention-orientated approaches is strongly emphasized in both national guidance (e.g. National Institute for Health and Care Guidance [11]) and international standards (e.g. ISO 45003 standards on psychological health and safety at work [14]), with reference to the central and ongoing role played by LMs throughout the process.

**Key learning points**

**What is already known about this subject:**

- Improving employee well-being is important for the overall performance of organizations.
- Line managers play a crucial role in supporting employees’ well-being and are strategically positioned to identify early signs of mental health issues.
- Providing line manager training in mental health is a recommended strategy for enhancing employee mental health and well-being.

**What this study adds:**

- Positive mental health and well-being practices increased throughout the COVID-19 pandemic—the proportion of organizations offering primary-, secondary- and tertiary-level interventions has increased year on year.
- Positive mental health and well-being practices cluster together—those organizations offering line manager training are more likely to offer a range of primary-, secondary- and tertiary-level interventions than organizations not offering this training.
- Among organizations offering line manager training in mental health, tertiary-level intervention activities are the most frequently adopted, followed by primary- and then secondary-level mental health and well-being practices.

**What impact this may have on practice or policy:**

- Organizations should invest in line manager training in mental health as part of their broader mental health strategy as a primary preventative initiative.
- Organizations should aim for a comprehensive approach that comprises the implementation of primary-, secondary- and tertiary-level mental health and well-being practices.
- Research is needed to quantify the specific impacts of line manager training on organizational-level outcomes, such as sickness absence and presenteeism.

LMs’ behaviours and wider management practices are a determinant of employee well-being [15–17]. It is, therefore, crucial to equip LMs with the knowledge, skills and abilities to (i) effectively support, guide and promote the MH&WB of their direct reports (people they manage); (ii) ensure they can design and manage people’s work to minimize work-related stress and (iii) cultivate a supportive and psychologically safe work environment. There is growing evidence that the necessary knowledge, skills and behavioural competencies needed to execute these tasks and roles by LMs can be learned and enhanced through targeted training programmes [18–20]. However, a survey conducted by the Institution of Occupational Safety and Health—prior to the COVID-19 pandemic—found that only 43% of organizations offered mental health training for their managers [21]. From 2020, the onset of the COVID-19 pandemic amplified workforce mental health risks [22]. While there are interventions being developed to support workforce mental health [23–26], the provision of LM training in mental health remains sub-optimal. Although data from large-scale employer surveys demonstrate that the proportion of organizations offering LM training has increased over time (to 59% in 2023), 41% of organizations still do not provide LM training in mental health [27].

There is little information available on the *context* in which LM training in mental health is delivered in organizations that provide it. The aim of this study, therefore, is to explore whether (or not) LM training initiatives contribute to a wider organizational strategy targeting employee well-being, which draws on a variety of workplace health promotion approaches and initiatives. To address this aim, the research question is: ‘Do organizations offering LM training differ in their adoption of primary-, secondary- and tertiary-level MH&WB practices, compared to those that do not?’.

## Methods

A secondary analysis of longitudinal, anonymized survey data from organizations in England was conducted. The data were derived from computer-assisted telephone interview surveys collected over 4 years, under a broader project ‘Mental health and well-being practices, outcomes, and productivity: A causal analysis’. Data were collected from employer representatives (business managers) in 2020 (1900 firms), 2021 (1551 firms), 2022 (1904 firms) and 2023 (1902 firms). Of these, 118 organizations participated in the survey for all 4 years. Throughout this study, the predictor variable was ‘LM training in mental health’, measured as a single, dichotomous variable (coded: no = 0, yes = 1). All outcome variables were measured as categorical variables. To explore the relationships between our predictor variable and outcomes, we conducted probit regression analyses to determine the probability of specific outcomes occurring based on the presence or absence of LM training in mental health. This allowed for a deeper understanding of how LM training relates to the use of other MH&WB practices by surveyed organizations. The analyses controlled for age of the organization (0–10 years, 11–20 years, more than 20 years), sector (Production, Construction, Wholesale/Retail, Hospitality, Business Services and Other Services) and size of the organization (micro/small:1–49; medium: 50–249; large: 250+ employees). The MH&WB practices offered by organizations were classified into primary, secondary and tertiary (File 1, available as Supplementary data at *Occupational Medicine* Online) as conceptually defined by the public health paradigm [28,29].

## Results

**Figure 1.**
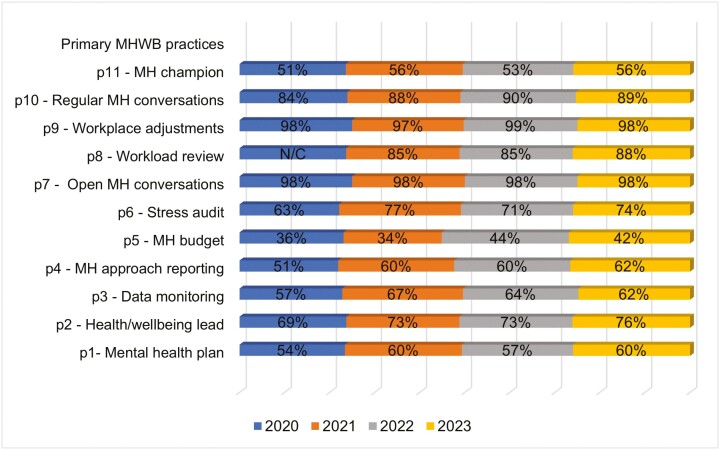
Primary-level intervention activities in organizations offering LM training in MH&WB (2020–2023).

We observed that organizations with LM training in mental health adopted more primary-level MH&WB practices compared to organizations without such training provisions (Table 1). This trend strengthened over the 4 years, evidenced by an increase in the proportions of firms offering LM training from 2020 to 2023 (2020: *n* = 413; 2021: *n* = 371; 2022: *n* = 497; 2023: *n* = 576) (Figure 1).

**Table 1.** Probit analysis of LM training in mental health associated with primary-level MH&WBs

DVs	2020 (826 firms)	2021 (838 firms)	2022 (962 firms)	2023 (963 firms)
P1. A mental health plan	β0.849*** LR chi^2^ 103.864 Log likelihood −122.561	β0.830*** LR chi^2^ 99.756*** Log likelihood −106.019	β0.738 LR chi^2^ 93.186 Log likelihood −128.806	β0.858*** LR chi^2^ 112.095 Log likelihood −127.528
P2. A health and well-being lead at Board or senior level	β0.757*** LR chi^2^ 77.303 Log likelihood −136.870	β0.593*** LR chi^2^ 68.612 Log likelihood −105.894	β0.722 LR chi^2^ 116.723 Log likelihood −133.211	β0.995*** LR chi^2^ 150.233 Log likelihood −117.462
P3. Use data to monitor employee health and well-being	β0.225* LR chi^2^ 42.222* Log likelihood −140.649	β0.574*** LR chi^2^ 72.360 Log likelihood −113.648	β0.422 LR chi^2^ 67.856 Log likelihood −133.239	β0.450*** LR chi^2^ 60.938 Log likelihood −133.995
P4. Internal and external reporting of your approach to mental health	β0.474*** LR chi^2^ 43.999 Log likelihood −135.150	β0.684*** LR chi^2^ 85.218 Log likelihood −114.934	β0.660 LR chi^2^ 86.506 Log likelihood −126.155	β0.712*** LR chi^2^ 92.692 Log likelihood −123.434
P5. A budget for mental health and well-being activities	β0.442*** LR chi^2^ 46.284 Log likelihood −123.069	β0.386*** LR chi^2^ 44.084 Log likelihood −102.342	β0.688*** LR chi^2^ 96.664 Log likelihood −125.725	β0.553*** LR chi^2^ 67.772 Log likelihood −119.435
P6. Risk assessments/stress audits	β0.242** LR chi^2^ 20.300* Log likelihood –127.340	β0.553 LR chi^2^ 52.740 Log likelihood −109.225	β0.454*** LR chi^2^ 45.863 Log likelihood −126.965	β0.593*** LR chi^2^ 63.998 Log likelihood −124.289
P7. Encourage open conversations about mental health in the workplace	β0.606** LR chi^2^ 20.707* Log likelihood −46.202	β0.605** LR chi^2^ 37.367*** Log likelihood −40.714	β0.721 LR chi^2^ 35.112 Log likelihood −57.725	β0.643 LR chi^2^ 37.389 Log likelihood −56.783
P8. Reviews of staff workloads	N/C	β0.412*** LR chi^2^ 38.946 Log likelihood −95.025	β0.454*** LR chi^2^ 48.828 Log likelihood −123.168	β0.457*** LR chi^2^ 44.623 Log likelihood −105.517
P9. Make appropriate workplace adjustments to those who need them to support their mental health	β0.324 LR chi^2^ 15.261 Log likelihood −47.421	β0.540 LR chi^2^ 21.291 Log likelihood −59.378	β0.901 LR chi^2^ 50.141 Log likelihood −49.353	β0.567*** LR chi^2^ 37.435 Log likelihood −58.642
P10. Ensure all staff have a regular conversation about their health and well-being with their manager	β0.342** LR chi^2^ 58.193 Log likelihood −112.132	β0.481*** LR chi^2^ 59.130 Log likelihood −91.725	β0.661 LR chi^2^ 110.301 Log likelihood −102.151	β0.459*** LR chi^2^ 55.004 Log likelihood −101.628
P11. Have employee mental health champions	β0.719*** LR chi^2^ 108.580 Log likelihood −117.790	β0.778*** LR chi^2^ 133.139 Log likelihood −123.329	β0.799*** LR chi^2^ 139.973 Log likelihood −122.047	β0.814*** LR chi^2^ 142.627 Log likelihood −123.649

N/C = not captured. LR chi^2^ = Likelihood ratio chi-square. Size, sector, and age of organizations are included as controls in all estimations.

*P < 0.05; **P < 0.01; ***P < 0.001.

**Figure 2.**
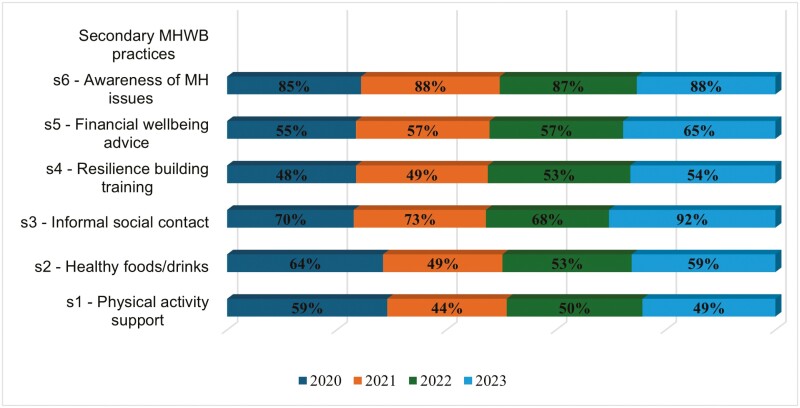
Secondary-level intervention activities in organizations offering LM training in MH&WB (2020–2023).

On average, organizations that offered LM training in mental health were more likely to adopt secondary-level MH&WB practices compared to organizations without LM training provisions (Table 2). Although the proportion of firms offering some of the secondary MH&WB practices increased from 2020 to 2023, there was an overall decrease in the proportion of firms offering general health promotion interventions including s1 ‘Support with physical activity such as gym memberships, cycle to work schemes’ and s2 ‘Supplying healthy food and drinks’ (Figure 2).

**Table 2.** Probit analysis of LM training in mental health associated with secondary-level MH&WBs

DVs	2020 (826 firms)	2021 (838 firms)	2022 (962 firms)	2023 (963 firms)
S1. Support with physical activity such as gym memberships, cycle to work schemes	β0.353*** LR chi^2^ 88.248 Log likelihood −117.339	β0.171 LR chi^2^ 68.051 Log likelihood −125.677	β0.333*** LR chi^2^ 97.958 Log likelihood −130.494	β0.315*** LR chi^2^ 77.704 Log likelihood −124.575
S2. Supplying healthy food and drinks	β0.192 LR chi^2^ 31.399 Log likelihood −118.948	β0.281** LR chi^2^ 76.003*** Log likelihood −127.599	β0.235*** LR chi^2^ 53.816 Log likelihood −131.633	β0.241** LR chi^2^ 69.302 Log likelihood −130.236
S3. Provide regular opportunities for informal social contact for remote workers	N/C	β0.213* LR chi^2^ 30.621** Log likelihood −112.107	β0.270** LR chi^2^ 68.297*** Log likelihood −122.070	β0.473* LR chi^2^ 10.909 (*P* = 0.365) Log likelihood −50.674
S4. Training aimed at building personal resilience	β0.632*** LR chi^2^ 81.347 Log likelihood −113.115	β0.721*** LR chi^2^ 99.676*** Log likelihood −119.168	β0.755*** LR chi^2^ 99.735 Log likelihood −123.847	β0.711 LR chi^2^ 93.078 Log likelihood −121.840
S5. Financial well-being advice	β0.444*** LR chi^2^ 32.414 Log likelihood −124.205	β0.358*** LR chi^2^ 62.422 Log likelihood −124.030	β0.496*** LR chi^2^ 68.844 Log likelihood −129.078	β0.556*** LR chi^2^ 86.178 Log likelihood −128.021
S6. Awareness raising for staff on mental health issues	β 1.109*** LR chi^2^ 161.334 Log likelihood −104.182	β0.939*** LR chi^2^ 125.462 Log likelihood −96.548	β0.946*** LR chi^2^ 167.966 Log likelihood −106.745	β 1.129*** LR chi^2^ 193.086 Log likelihood −108.650

N/C = not captured. DVs = dependent variables. LR chi^2^ = Likelihood ratio chi-square.

*P < 0.05; **P < 0.01; ***P < 0.001.

**Figure 3.**
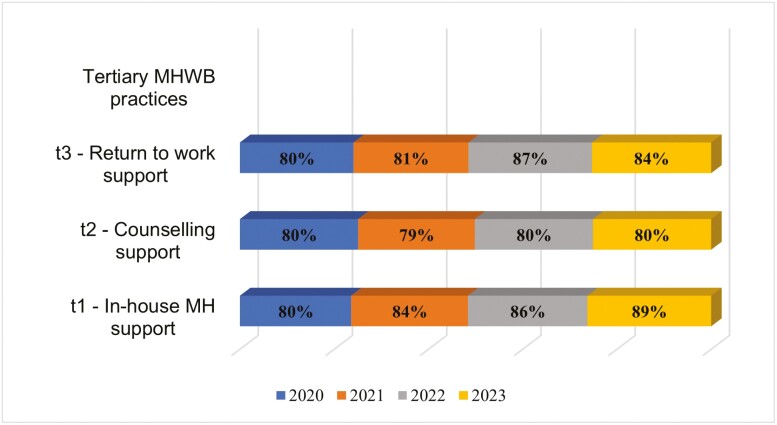
Tertiary-level intervention activities in organizations offering LM training in MH&WB (2020–2023).

On average, organizations that offered LM training in mental health were more likely to adopt tertiary-level MH&WB practices compared to organizations without LM training provisions (Table 3). The proportion of firms offering these activities also increased from 2020 to 2023 or 2021 to 2023, where applicable (Figure 3).

**Table 3.** Probit analysis of LM training in mental health associated with tertiary-level MHWBs

DVs	2020 (826 firms)	2021 (838 firms)	2022 (962 firms)	2023 (963 firms)
T1. In-house MH support and signposting to other services	β0.784*** LR chi^2^ 99.356 Log likelihood −102.512	β0.606*** LR chi^2^ 104.804 Log likelihood −98.702	β0.829*** LR chi^2^ 139.387 Log likelihood −106.141	β0.980*** LR chi^2^ 157.511 Log likelihood −93.751
T2. Access to counselling support	N/C	β0.572*** LR chi^2^ 87.235 Log likelihood −109.354	β0.635*** LR chi^2^ 101.919 Log likelihood −115.418	β0.525*** LR chi^2^ 87.867 Log likelihood −102.585
T3. Training and support for those returning to work	N/C	β0.465*** LR chi^2^ 58.564 Log likelihood −113.247	β0.732*** LR chi^2^ 112.463 Log likelihood −111.429	β0.741 LR chi^2^ 108.555 Log likelihood −107.213

N/C = not captured. DVs = dependent variables. LR chi^2^ = Likelihood ratio chi-square.

*P < 0.05; **P < 0.01; ***P < 0.001.

Finally, the average proportions of primary-, secondary- and tertiary-level MH&WB practices were computed to determine which intervention levels were most adopted by organizations over the years. Overall, tertiary-level MH&WB practices were most adopted (2020: 80%; 2021: 81%; 2022: 84%; 2023: 84%), followed by primary-level MH&WB practices (2020: 66%; 2021: 72%; 2022: 72%; 2023: 73%) and then, secondary-level MH&WB practices (2020: 62%; 2021: 60%; 2022: 61%; 2023: 67%).

## Discussion

This study explored the relationship between LM training in mental health and the adoption of primary-, secondary- and tertiary-level MH&WB practices. Our findings show that LM training was significantly associated with MH&WB practices across all three levels. For organizations that offer LM training, there was a consistent increase in the proportion of primary- (prevention-focused) and tertiary (curative/remedial)-level MH&WB practices offered across the 4 years. However, there was some variation in the proportion of secondary-level MH&WB practices offered, with some increasing and others, in contrast, decreasing over time. Among the three intervention levels, tertiary interventions were adopted most frequently, followed by primary and then secondary interventions.

A strength of this study is that it provides a comprehensive analysis of how a large sample of UK organizations adapted their MH&WB practices over several years, from immediately before (January 2020), to the end (May 2023) of a pandemic. The focus on LM training in mental health presents a valuable contribution by highlighting the overall benefits of providing this training to the wider organizational customs and practices. To the best of our knowledge, this is the first study to examine the relationship between LM training in mental health and the broader use of primary-, secondary- and tertiary-level MH&WB practices by organizations. However, the use of unbalanced panel data in the analyses limits our ability to capture the genuine ‘longitudinal’ effects of LM training on the organizational adoption of MH&WB practices. Due to the variations in the number of observations at each time point, there is reduced precision in capturing the temporal dynamics of the relationships being investigated. While our measures capture the presence/absence of various MH&WB practices (including LM training) and demonstrate how they are related, further research is required to determine the effectiveness of these practices on individual- and organizational-level outcomes.

Our study contributes to the growing body of literature which highlights the importance of providing mental health training for LMs in the workplace [15,18,20,29]. While current intervention studies are exploring the impacts of LM training on individual employees and their LMs (e.g. Total Worker Health Intervention [25]; Managing Minds at Work [23,26]), our study explores patterns of well-being intervention at an organizational level which, to our knowledge, have not previously been documented. The establishment of a relationship between the provision of LM training in mental health and other positive MH&WB policies and practices suggests that the training of LMs in mental health is associated with a broader organizational commitment to employee well-being at all three intervention levels. Essentially, we observed that positive MH&WB practices cluster together. Further research is needed to explore the types of intervention (i.e. their content/nature, dose, duration and frequency) that are more, or less, effective for improving workforce well-being and indices of business performance.

The fact that we identified increases in the adoption of MH&WB practices over recent years is promising given the rise in mental health problems in working adults during and after the pandemic [30]. This suggests a greater awareness of employers relating to mental health at work, which manifests in actions to mitigate or manage this growing trend. The association between the provision of LM training and other MH&WB practices perhaps indicates that raising managers’ awareness, knowledge, confidence and skills relating to workforce mental health may act as a catalyst for the implementation of positive, health-focused practices across the organization.

A notable finding from this study is the increasing proportions of tertiary-level interventions used by organizations over the 4 years. Previous research suggests that organizations may opt for tertiary-level interventions due to the perceived immediate benefits and tangibility of support services [31]. However, while these interventions are important in offering support to employees already suffering from mental health issues, their effects are not as long-lasting as primary and secondary interventions—as they do not address the root causes of the issue [32]. Hence, scholars argue that the best approach for addressing mental health issues at work (e.g. work-related stress) is a balanced, holistic approach that combines all three intervention levels [33]. Future research in this area should focus on quantifying the specific impacts of LM training on organizational-level outcomes, such as sickness absence and presenteeism. This evidence would help to inform employers’ investment decisions relating to MH&WB at work, which will ultimately impact employee health and well-being.

## Supplementary Material

kqae051_suppl_Supplementary_Material
