# Supplementary material for: Line manager training and organizational approaches to supporting well-being
Source: Occup Med (Lond). 2024 Jul 11;74(6):416–22. doi: 10.1093/occmed/kqae051 (PMC11419705; doi:10.1093/occmed/kqae051)
Supplement: kqae051_suppl_Supplementary_Material [file kqae051_suppl_supplementary_material.zip › Supplementary File 1.docx]

Supplementary file 1. Categorisation of Mental Health and Wellbeing Practices by Intervention Level.

| **Primary** | **Secondary** | **Tertiary** |
| --- | --- | --- |
| A mental health plan | Support with physical activity such as gym memberships, cycle to work schemes | In-house MH support and signposting to other services |
| Use data to monitor employee health and wellbeing | Supplying healthy food and drinks | Access to counselling support |
| Internal and external reporting of your approach to MH | Provide regular opportunities for informal social contact for remote workers | Training and support for those returning to work |
| A budget for MH and wellbeing activities | Training aimed at building personal resilience |  |
| Risk assessments/stress audits | Financial wellbeing advice |  |
| Reviews of staff workloads | Awareness raising for staff on MH issues |  |
| Encourage open conversations about mental health in the workplace |  |  |
| Make appropriate workplace adjustments to those who need them to support their mental health |  |  |
| Ensure all staff have a regular conversation about their health and wellbeing with their manager.  Have employee mental health champions. |  |  |
